# Deterministic entanglement-assisted quantum communication over 20 km fiber channel

**DOI:** 10.1038/s41377-025-02173-6

**Published:** 2026-01-23

**Authors:** Siyu Ren, Yanru Yan, Yalin Li, Chao Li, Dongmei Han, Xuezhi Zhu, Meihong Wang, Xiaolong Su

**Affiliations:** 1 State Key Laboratory of Quantum Optics Technologies and Devices, Institute of Opto-Electronics, Shanxi University, Taiyuan, China; 2 Collaborative Innovation Center of Extreme Optics, Shanxi University, Taiyuan, China

**Keywords:** Quantum optics, Quantum optics

## Abstract

Entanglement-assisted quantum communication has substantial advantages in surpassing the power of classical communication by utilizing the entangled state. Up to now, most of entanglement-assisted quantum communications with dense coding are limited to the proof-of-principle experiments. Here, we experimentally demonstrate the deterministic entanglement-assisted quantum communication based on the continuous-variable (CV) entangled state over 20 km commercial fiber channels. We propose a new CV dense coding scheme with improved classical signals and show that the transmission distance of CV entanglement-assisted quantum communication can be extended compared with that using fixed classical signals. By applying the frequency division multiplexing technique, we simultaneously decode 10 classical signals submerged in the shot noise of coherent state with the help of CV entangled state after the transmission through a 20.121 km fiber channel. The results show that around 3 times of channel capacity in classical communication with coherent state are achieved in the CV entanglement-assisted communication with the frequency division multiplexing technique. Our result takes a crucial step towards realizing the deterministic metropolitan entanglement-assisted quantum communication in practical quantum channels.

## Abstract (web summary)

We experimentally demonstrate the deterministic entanglement-assisted quantum communication based on the continuous-variable entangled state over 20 km fiber channel.

## Graphical abstract

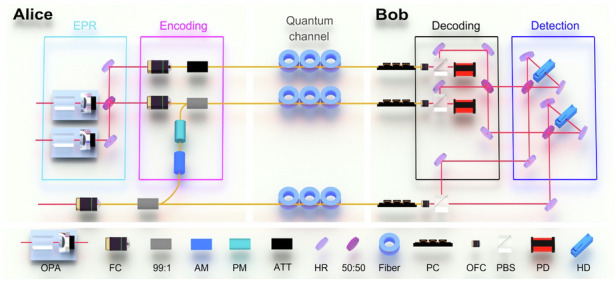

## Introduction

Quantum entanglement, which serves as an important quantum source, is widely used in quantum communication^1–7^, quantum computing^8–10^, and quantum sensing^11–13^. As a typical entanglement-assisted quantum communication (EAQC) protocol, quantum dense coding enables two communication parties to enhance the channel capacity with the shared quantum entanglement^14^. In quantum dense coding, two classical bits are encoded on one quantum bit from an Einstein-Podolsky-Rosen (EPR) pair or beam, and therefore the capacity of a quantum channel is doubled if the particle or beam is transmitted and all messages are retrieved^14^. Besides the advantage of enhancing the channel capacity in quantum communication, quantum dense coding has potential advantages in quantum key distribution^15–20^, since the signal-to-noise ratio (SNR) is improved due to the lower noise background of the correlated noise for entangled state. In addition, quantum dense coding can also be used in quantum metrology^21^ since two non-commuting observables are measured simultaneously.

Since the first experimental demonstration of quantum dense coding with entangled photon pairs^22^, it has been experimentally demonstrated in several physical systems, including optical system^23–29^, nuclear magnetic resonance system^30^, and atomic system^31–33^. Various entangled states, including spin and orbital angular momentum entangled state^28^, orbital angular momentum multiplexed entangled state^33^ and high-dimensional entangled state^27^ have been applied to improve the channel capacity in EAQC with dense coding. Besides the point-to-point quantum communication, quantum dense coding has also been extended to network communication with multipartite entangled states, where dense coding between two parties is controlled by other communication parties^34–37^.

Compared with quantum dense coding with the discrete-variable (DV) system, the deterministic quantum dense coding can be achieved with the continuous-variable (CV) system since the generation of Gaussian entangled state and the detection system are deterministic^38–41^. The deterministic quantum dense coding allows signal transmission with high efficiency^42^, in contrast to the low efficient transmission in DV systems caused by the probabilistic generation of entangled photons through weak parametric down-conversion^7^. Besides, the CV quantum dense coding system is compatible with the current optical communication system, since the way to encode classical signals and the detector used in the CV scheme are widely used in classical optical communication systems. In the CV quantum dense coding, the classical signals encoded on both amplitude and phase quadratures of one EPR entangled beam are decoded simultaneously with the help of the other EPR beam. The channel capacity is enhanced since both correlated noise of amplitude and phase quadratures of EPR beams are lower than the shot noise limit (SNL), which leads to the increase of SNRs of two classical signals. However, most demonstrations of EAQC with dense coding still remain in proof-of-principle experiments, i.e. the transmission distances are limited to meter scale.

Here, we experimentally demonstrate the deterministic EAQC based on the CV entangled state in the fiber channel. Compared to previous proof-of-principle experiments, we extend the transmission distance of deterministic EAQC from meters to 20.121 km, which has potential applications in the metropolitan quantum communication. The EAQC over 20 km is achieved by applying the improved classical signals considering the transmission efficiency in the encoding process and transmitting the EPR entangled beams and local beam through independent fibers, which reduces the excess noise added to the EPR entangled beams to about 0.01 shot noise unit (SNU). We show that the measured SNRs of weak encoded signals are improved by applying CV entangled state and the channel capacity in the EAQC is higher than that with the coherent state in 0.002, 2.017, 5.043, 10.074, and 20.121 km fiber channels, respectively. Furthermore, the channel capacity of the EAQC is enhanced significantly by the frequency division multiplexing (FDM). Our results demonstrate the feasibility of the deterministic EAQC in the fiber channel and take a key step toward metropolitan quantum communication.

## Results

### The principle

**Fig. 1:**
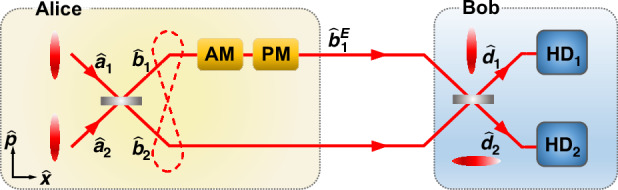
The principle of the deterministic EAQC with dense coding. Alice encodes classical signals on the amplitude and phase quadratures of one of the CV EPR entangled beams by an amplitude modulator (AM) and a phase modulator (PM) and distributes the EPR entangled beams to Bob. After receiving the transmitted EPR entangled beams, Bob performs the joint measurement by coupling two beams on a 50:50 beamsplitter and measuring the output states with two homodyne detectors (HDs) simultaneously. In this way, the classical signals encoded on the amplitude and phase quadratures are decoded simultaneously

As shown in Fig. 1, the CV EPR entangled state with optical modes ${\hat{b}}_{1}$ and ${\hat{b}}_{2}$, which are used as encoded and decoding modes in the CV quantum dense coding respectively^24^, is obtained by coupling two amplitude squeezed states ${\hat{a}}_{1}$ and ${\hat{a}}_{2}$ on a 50:50 beamsplitter. In the encoding process, Alice encodes classical signals ${\hat{x}}_{s}$ and ${\hat{p}}_{s}$ on the amplitude and phase quadratures of the optical mode ${\hat{b}}_{1}$ simultaneously. The amplitude and phase quadratures of the encoded mode are given by ${\hat{x}}_{{b}_{1}^{E}}={\hat{x}}_{{b}_{1}}+{\hat{x}}_{s}$ and ${\hat{p}}_{{b}_{1}^{E}}={\hat{p}}_{{b}_{1}}+{\hat{p}}_{s}$ respectively. After the encoding process, Alice sends both optical modes ${\hat{b}}_{1}^{E}$ and ${\hat{b}}_{2}$ to Bob through quantum channels. In the decoding process, Bob couples the encoded mode ${\hat{b}}_{1}^{E}$ and decoding mode ${\hat{b}}_{2}$ on a 50:50 beamsplitter and obtains two displaced squeezed states ${\hat{d}}_{1}$ and ${\hat{d}}_{2}$, whose amplitude and phase quadratures are measured by two homodyne detectors, respectively. In this way, the correlated EPR noise of the encoded and decoding modes are canceled and the encoded classical signals of ${\hat{x}}_{s}$ and ${\hat{p}}_{s}$ are retrieved simultaneously with the noise background of squeezed noise, which is below the SNL corresponding to the noise of vacuum state (see Materials and Methods for details).

The performance of deterministic EAQC can be quantified by the channel capacity. For the communication in a channel with Gaussian noise, the Shannon channel capacity is expressed by $C=\frac{1}{2}{\log }_{2}[1+SNR]$^41^. Since the encoded amplitude and phase signals are decoded simultaneously, the channel capacity of the deterministic EAQC is given by

1 $$C=\frac{1}{2}{\log }_{2}[1+SN{R}_{x}]+\frac{1}{2}{\log }_{2}[1+SN{R}_{p}]$$

where *SNR*_*x*_ and *SNR*_*p*_ represent the SNRs of amplitude and phase signals respectively. Based on the expression of output states at Bob’s station [Eq. (8) in Materials and Methods], we obtain SNRs of amplitude and phase signals, which are given by

2 $$\begin{array}{rcl}SN{R}_{x} & = & \frac{\frac{1}{2}V\left({\hat{x}}_{s}\right)}{V\left({\hat{x}}_{{a}_{1}}\right)}\\ SN{R}_{p} & = & \frac{\frac{1}{2}V\left({\hat{p}}_{s}\right)}{V\left({\hat{x}}_{{a}_{2}}\right)}\end{array}$$

where $V({\hat{x}}_{s})$ and $V({\hat{p}}_{s})$ are the variances of modulated classical signals, the coefficient 1/2 indicates that the signals are splitted by the 50:50 beamsplitter during the decoding process (see Materials and Methods for details).

When the EPR entangled state is replaced by two coherent states ${\hat{c}}_{1}$ and ${\hat{c}}_{2}$, the noise background of the demodulated signals are $V({\hat{x}}_{{c}_{1}})=V({\hat{x}}_{{c}_{2}})=1$ respectively, which is the same as SNL. In this case, if the weak classical signals with variance of $V({\hat{x}}_{s})=V({\hat{p}}_{s})=2$ (two times of SNL) are encoded by Alice, the variances of the decoded classical signals are $\frac{1}{2}V({\hat{x}}_{s})=\frac{1}{2}V({\hat{p}}_{s})=1$ at Bob’s station, which are submerged in the noise of the coherent state since *SNR*_*x*_ = *SNR*_*p*_ = 1. When the EPR entangled state is used as the quantum resource, the encoded weak classical signals will be retrieved simultaneously with *SNR*_*x*_ = *SNR*_*p*_ > 1, which embodies the advantage of deterministic EAQC, since the noise background $V({\hat{x}}_{{a}_{1}})=V({\hat{x}}_{{a}_{2}})={e}^{-2r}$ is reduced below the SNL when *r* > 0 in Eq. (2).

Considering the transmission in the fiber channel, the received modes at Bob’s station turn into ${\widehat{b}}_{1}^{{E}^{{\prime} }}=\sqrt{\eta }{\hat{b}}_{1}^{E}+\sqrt{1-\eta }({\hat{\nu }}_{1}+{\hat{\zeta }}_{1})$ and ${\hat{b}}_{2}^{{\prime} }=\sqrt{\eta }{\hat{b}}_{2}+\sqrt{1-\eta }({\hat{\nu }}_{2}+{\hat{\zeta }}_{2})$ respectively, where *η* = *η*_*d*_*η*_*t*_*η*_*c*_ represents the total efficiency of the system, *η*_*d*_ represents the detection efficiency, *η*_*t*_ = 10^−0.2*L*/10^ represents the transmission efficiency in the fiber channel with transmission distance *L*, *η*_*c*_ represents the transmission efficiency of other fiber devices, ${\hat{\nu }}_{i}$ represents the vacuum state introduced by the loss, and ${\hat{\zeta }}_{i}$ is the excess noise with the variance of *δ*_*i*_ in the fiber channel. In this case, the SNRs at Bob’s station are given by

3 $$\begin{array}{rcl}SN{R}_{x}^{{\prime} } & = & \frac{\frac{1}{2}\eta V\left({\hat{x}}_{s}\right)}{\eta V\left({\hat{x}}_{{a}_{1}}\right)+\frac{1}{2}(1-\eta )[V\left({\hat{x}}_{{\nu }_{1}}\right)+V\left({\hat{x}}_{{\nu }_{2}}\right)+{\delta }_{1}+{\delta }_{2}]}\\ SN{R}_{p}^{{\prime} } & = & \frac{\frac{1}{2}\eta V\left({\hat{p}}_{s}\right)}{\eta V\left({\hat{x}}_{{a}_{2}}\right)+\frac{1}{2}(1-\eta )[V({\hat{p}}_{{\nu }_{1}})+V({\hat{p}}_{{\nu }_{2}})+{\delta }_{1}+{\delta }_{2}]}\end{array}$$

where $V({\hat{x}}_{{\nu }_{1}})=V({\hat{x}}_{{\nu }_{2}})=V({\hat{p}}_{{\nu }_{1}})=V({\hat{p}}_{{\nu }_{2}})=1$ are the variances of the vacuum states (see Materials and Methods for details).

**Fig. 2:**
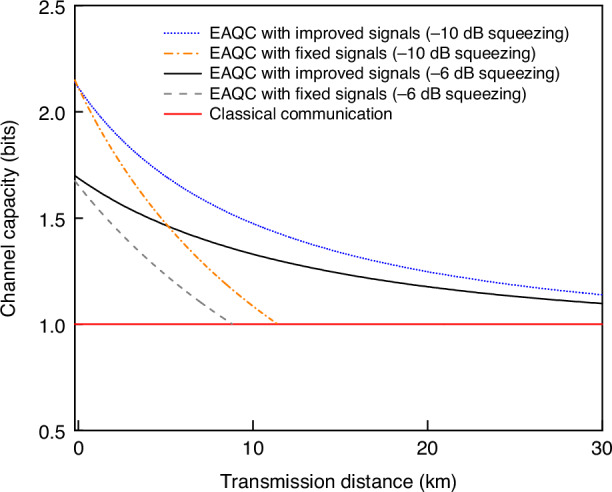
Theoretical prediction for the dependence of channel capacity on transmission distance with different encoded classical signals. The transmission distance with improved classical signals is increased compared with that using fixed classical signals at Alice’s station. The black solid and gray dashed curves are obtained with the squeezing (antisqueezing) level of −6 dB (+10 dB), and the blue dotted and orange dot-dashed curves are obtained with the squeezing (antisqueezing) level of −10 dB (+15 dB). Phase fluctuation is not considered here

The SNRs and channel capacity decrease with the decrease of transmission efficiency according to Eqs. (1) and (3). By substituting Eq. (3) into Eq. (1), we theoretically predict the achievable channel capacity and transmission distance of deterministic EAQC based on quantum dense coding, as shown in Fig. 2. When fixed classical signals (${V}_{f}({\hat{x}}_{s})={V}_{f}({\hat{p}}_{s})=2$) are encoded at Alice’s station, i.e. the amplitude of encoded signals are fixed, the transmission distance is limited to 10 km when the channel capacity of EAQC is higher than that of coherent state, as shown by the gray dashed curve in Fig. 2. In order to extend the transmission distance, we propose a new encoding method, where Alice improves the encoded signals to ${V}_{i}({\hat{x}}_{s})={V}_{i}({\hat{p}}_{s})=2/\eta$ by considering the effect of transmission efficiency, which can be regarded as normalizing the encoded classical signals to the sender (Alice). In this case, the variances of decoded signals at Bob’s station in Eq. (3) turn into $\frac{1}{2}\eta {V}_{i}({\hat{x}}_{s})=\frac{1}{2}\eta {V}_{i}({\hat{p}}_{s})=1$, which means that the retrieved signals are just submerged in the SNL at different transmission distance. The simulation results show that the transmission distance can be extended over 30 km with improved classical signals, where the channel capacity of EAQC is still higher than that of coherent state, as shown by the black solid curve in Fig. 2. When the squeezing (antisqueezing) level is increased to -10 dB (+15 dB), it is obvious that the channel capacity and transmission distance can be further increased. Thus, we choose the improved classical signals in the encoding process in our experiment.

### The experiment

**Fig. 3:**
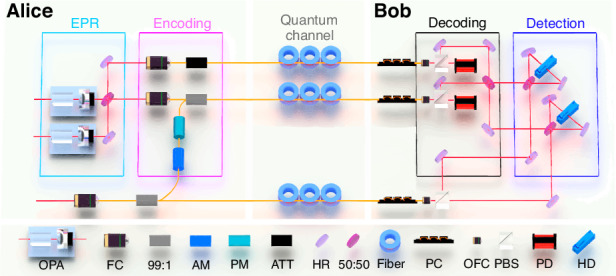
Schematic of the experimental setup. The EPR entangled beams generated from two squeezed states are coupled into commercial fibers (SM-28e) by two high precision fiber couplers (FC) respectively. A coherent beam is also coupled into a fiber and divided into two parts, which are used as the ancilla beam and the local beam respectively. Alice encodes classical signals on the ancilla beam by an amplitude modulator (AM, MXER-LN-10) and a phase modulator (PM, MPX-LN-0.1) and couples it with one of the EPR entangled beams by a 99:1 fiber beamsplitter (PMTC-55-2-01-2-B-P-Q-F). An attenuator (ATT) is used to balance the loss between two fiber channels due to the inherent loss of fiber devices such as fiber piezo and fiber beam splitter. Both the entangled beams and local beam are transmitted to Bob through fiber channels respectively. At Bob’s station, the combination of a polarization controller (PC), a polarization beam splitter (PBS) and a photodetector (PD) is used to monitor and control the polarization of output beams from fiber channels, which offers a deviation of about 2% in several minutes and it is enough for the data measurement. The entangled beams and the local beam are coupled to free space after transmission by three optical fiber collimators (OFC) and the output beams after decoding are measured by two HDs. The details of the laser source can be found in Materials and Methods

In the experiment, two squeezed states at 1550 nm are generated from two optical parametric amplifiers (OPAs) respectively, as shown in Fig. 3. The OPA, which is a semi-monolithic cavity, consists of a periodically poled potassium titanyl phosphate (PPKTP) crystal and a concave mirror (see Materials and Methods for details). When the OPA is operated in the de-amplification status, where the relative phase between the pump beam and seed beam is controlled to *π*, an amplitude squeezed state with the squeezing (antisqueezing) level of − 6 (+ 10) dB is prepared. By coupling two squeezed states on a 50:50 beamsplitter with the relative phase of *π*/2, the CV EPR entangled state is obtained. Two optical modes of the EPR entangled state are coupled into the fiber by two high-precision fiber couplers with the coupling efficiency of 96%, respectively.

In the encoding process, Alice modulates classical signals on an ancilla coherent beam simultaneously by the fiber amplitude and phase modulators, respectively, and couples the modulated ancilla beam with the encoded mode ${\hat{b}}_{1}$ by a 99:1 fiber beamsplitter. The classical signals are all simulated cosine signals generated from the signal generators. Alice sends two classical signals with frequencies of 8 MHz (serving as the amplitude signal) and 8.5 MHz (serving as the phase signal) to Bob, respectively. Both the encoded mode ${\hat{b}}_{1}^{E}$ (carrying classical signals) and the decoding mode ${\hat{b}}_{2}$ are transmitted to Bob through two independent fiber channels. In the decoding process, two received optical modes are coupled on a 50:50 beamsplitter with the relative phase difference of 0 at Bob’s station. The amplitude and phase quadratures of two output states of the beamsplitter are measured by two homodyne detectors with the help of the local beam to extract the encoded classical signals simultaneously. The local beam of the homodyne detector, which is obtained from a part of Alice’s ancilla coherent beam, is also transmitted to Bob through another independent fiber channel.

### Experimental results

**Fig. 4:**
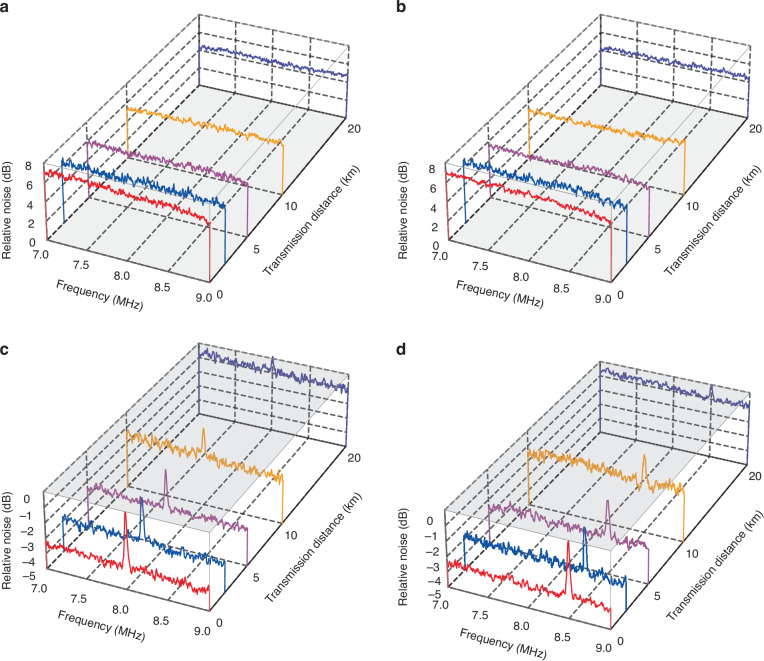
Measurement results of the deterministic EAQC in the fiber channel. **a**, **b** Power spectra for the amplitude and phase quadratures of EPR noise measured by Bob at different transmission distances. **c**, **d** Power spectra for the amplitude and phase signals decoded by Bob at different transmission distances. The red, blue, pink, orange, and purple curves represent the measurement results at the transmission distances of 0.002, 2.017, 5.043, 10.074, and 20.121 km, respectively. The gray plane represents the SNL. The power spectra are recorded with a spectrum analyzer, where the resolution bandwidth of 30 kHz and video bandwidth of 100 Hz are chosen, and each curve is averaged 10 times

Firstly, we measure the amplitude and phase quadratures of the encoded mode at Bob’s station by a homodyne detector, where the decoding mode is not involved. In this case, only the EPR noise is observed, which are around 7.23 (6.92), 6.24 (6.44), 5.56 (5.70), 5.34 (5.10), and 4.45 (4.51) dB higher than the SNL for the amplitude (phase) quadrature at transmission distances of 0.002, 2.017, 5.043, 10.074 and 20.121 km respectively, as shown in Fig. 4a (Fig. 4b). It is obvious that the encoded classical signals are not observed since the weak modulated signals are completely submerged in the EPR noise. The measurement results also show that the EPR noise decreases with the increase of the transmission distance due to the increased loss in the fiber channel.

Secondly, we decode the classical signals with the help of the decoding mode. As shown in Fig. 4c and d, the measurement results show that the classical signals are retrieved simultaneously with the help of the EPR entangled state in the deterministic EAQC. In this case, the classical amplitude signal at 8 MHz is retrieved from the amplitude squeezed state ${\hat{d}}_{1}$, where the noise are around 3.71, 3.02, 2.61, 1.80, and 0.92 dB below the SNL at the corresponding transmission distances of 0.002, 2.017, 5.043, 10.074, and 20.121 km respectively (Fig. 4c). The classical phase signal at 8.5 MHz is also retrieved simultaneously from the phase squeezed state ${\hat{d}}_{2}$, where the noise are around 3.63, 3.11, 2.59, 1.73, and 0.96 dB below the SNL at five transmission distances (Fig. 4d). The peaks of the retrieved signals just reach the noise of SNL, which means that the weak modulated classical signals at Alice’s station can not be retrieved with the coherent state since the noise level of it is the same as the SNL. The results in Fig. 4c and d demonstrate that the weak classical signals encoded by Alice can always be retrieved as long as the noise background at Bob’s station is lower than the SNL by using the improved classical signals.

The performance of the deterministic EAQC is affected by the total efficiency, excess noise, and phase fluctuation, as shown in Table 1. By replacing the entangled state with the coherent state that has the same power, the total efficiency and excess noise are estimated via measuring the power and variance of the coherent state before and after the transmission through the fiber channel, respectively. The estimated excess noises of the fiber channel for the transmission of EPR entangled state at different transmission distances are about 0.01 SNU. In our experiment, the most challenging part is the phase fluctuation at Bob’s station, which comes from optical beams after the transmission in independent fiber channels. After optimizing the phase locking system, phase fluctuations at transmission distances of 0.002, 2.017, 5.043, 10.074 and 20.121 km are reduced to 1.01°, 1.52°, 1.70°, 2.21°, and 2.45° respectively. The details of the locking system and the phase fluctuation can be found in Supplementary Information.

**Table 1:** Experimental parameters and the measured signal-to-noise ratios

Distance (km)	Total efficiency	Excess noise	Phase fluctuation	*SNR*_*x*_ (dB)	*SNR*_*p*_ (dB)
0.002	78%	0.007	1.01°	3.71	3.63
2.017	71%	0.010	1.52°	3.02	3.11
5.043	62%	0.011	1.70°	2.61	2.59
10.074	49%	0.013	2.21°	1.80	1.73
20.121	31%	0.016	2.45°	0.92	0.96

**Fig. 5:**
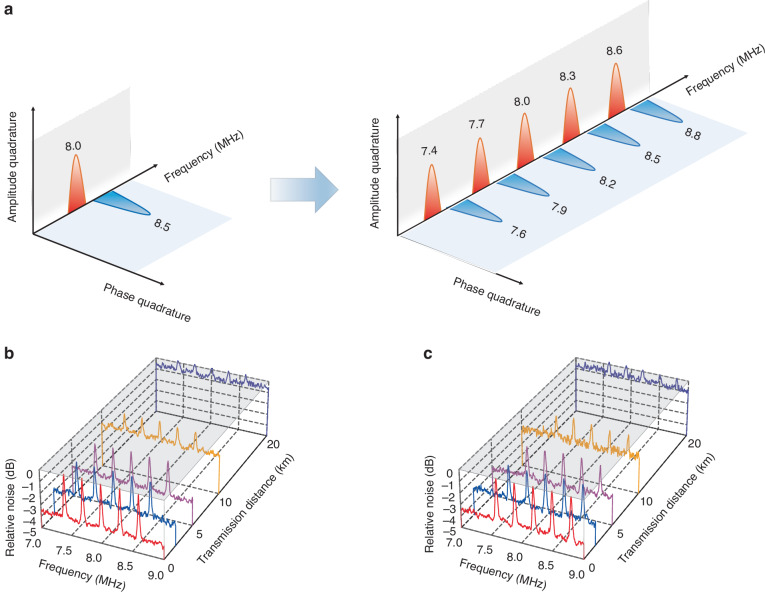
Results of deterministic EAQC combined with the FDM technique. **a** The schematic of encoded classical signals at different frequencies utilizing FDM technique. **b** and **c** Power spectra for the decoded amplitude and phase signals measured by Bob at different transmission distances. The red, blue, pink, orange, and purple curves represent the measurement results for 5 amplitude and 5 phase signals at different transmission distances. The gray plane represents the SNL. The power spectra are recorded with a spectrum analyzer, where the resolution bandwidth of 30 kHz and video bandwidth of 100 Hz are chosen, and each curve is averaged 10 times

Furthermore, we apply the FDM technique to improve the channel capacity of the deterministic EAQC. In this case, the encoded amplitude signal at 8.0 MHz and phase signal at 8.5 MHz are extended to 5 amplitude classical signals at 7.4, 7.7, 8.0, 8.3, 8.6 MHz and 5 phase classical signals at 7.6, 7.9, 8.2, 8.5, 8.8 MHz respectively, as shown in Fig. 5a. With the help of the decoding beam, 5 amplitude and 5 phase signals are retrieved simultaneously at transmission distances of 0.002, 2.017, 5.043, 10.074, and 20.121 km respectively, as shown in Fig. 5b and c. The noise backgrounds are all below the SNL due to the existed entanglement at different transmission distances. The detailed SNRs of these 10 signals can be found in Table 2.

**Table 2:** The measured signal-to-noise ratios with FDM

Distance (km)	*SNR*_*x*_ (dB)					*SNR*_*p*_ (dB)				
	7.4 MHz	7.7 MHz	8.0 MHz	8.3 MHz	8.6 MHz	7.6 MHz	7.9 MHz	8.2 MHz	8.5 MHz	8.8 MHz
0.002	3.67	3.73	3.65	3.69	3.72	3.62	3.61	3.65	3.61	3.70
2.017	2.88	2.85	2.95	2.90	2.87	2.89	3.08	3.10	3.08	3.02
5.043	2.55	2.67	2.71	2.69	2.64	2.59	2.63	2.59	2.66	2.58
10.074	1.57	1.63	1.66	1.67	1.65	1.62	1.65	1.62	1.67	1.69
20.121	0.95	0.88	0.94	0.87	0.88	0.81	0.83	0.83	0.91	0.93

**Fig. 6:**
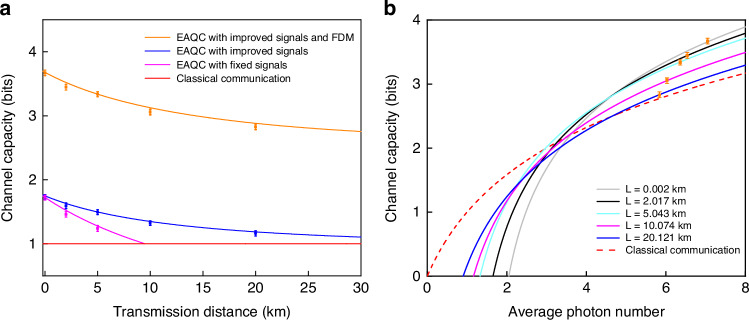
The channel capacity of EAQC based on dense coding. **a** The dependence of channel capacity on transmission distance. The channel capacity of EAQC with improved signals and FDM (orange solid) is increased compared to that without FDM (blue solid), and they are all higher than that with fixed classical signals (pink solid) and classical communication using coherent state with and without FDM (red solid). **b** The dependence of channel capacity on average photon number. The channel capacities of EAQC based on quantum dense coding at the transmission distances of 0.002 (gray solid), 2.017 (black solid), 5.043 (cyan solid), 10.074 (pink solid), and 20.121 (blue solid) km are higher than that of classical communication with coherent state (red dashed) in the case of large average photon number

Finally, we compare the channel capacity of the EAQC with that of the coherent state. According to SNRs obtained from the measured noise power spectra in Figs. 4c, d, 5b and c, we obtain the channel capacity of 3.66, 3.44, 3.35, 3.05, and 2.83 (1.73, 1.59, 1.49, 1.32, and 1.16) for EAQC with (without) FDM at transmission distances of 0.002, 2.017, 5.043, 10.074, and 20.121 km respectively, as shown in Fig. 6a (The SNRs of deterministic EAQC with fixed classical signals at different transmission distances can be found in Supplementary Information). When the entangled state is replaced by the coherent state, the channel capacity equals to 1, which means that the improved weak classical signals are just submerged in the noise background of coherent state with heterodyne detection. Compared to the communication with coherent state in the same condition, the channel capacity of deterministic EAQC with FDM technique is enhanced significantly.

We also obtain the dependence of channel capacity on average photon number, as shown in Fig. 6b (The detailed analysis can be found in Supplementary Information). Increasing the average photon number is beneficial to the enhancement of channel capacity. Compared to dense coding with the coherent state, EAQC based on quantum dense coding exhibits the advantage of enhancing the channel capacity at large average photon number. When the FDM is applied in our experiment, although the channel capacity with entangled state decreases with the increase of transmission distance, they are all higher than that of coherent state at the same average photon number, as shown by the orange points in Fig. 6b. Our results demonstrate the advantage of EAQC based on quantum dense coding in enhancing the channel capacity in the fiber channel.

## Discussion

Toward practical application of the presented deterministic EAQC, it is essential to further improve the transmission distance and channel capacity. The transmission distance and channel capacity can be increased by decreasing the channel loss and increasing the initial squeezing level of the squeezed state. For example, the transmission distance with channel capacity of 1.16 bits can be extended to 35 km when the ultra-low-loss fiber^43^ with attenuation of 0.14 dB/km and initial squeezing of 10 dB are applied. Besides, increasing the number of FDM provides another efficient method to increase the channel capacity of CV EAQC. In this case, the broadband squeezed light is required, since the number of FDM is limited by the bandwidth of squeezed light. In addition, the channel capacity can also be increased via increasing the amplitude of classical signals.

Since the channel capacity is enhanced with dense coding, the data rate of EAQC with the binary phase-shift keying (BPSK) modulation can be increased compared with that of the classical communication^42^. The demonstrated EAQC with dense coding in the fiber channel is beneficial to secure quantum communication, for example, it can be applied to dense quantum key distribution^15,44^. In CV dense quantum key distribution, the secret key can be obtained by replacing the encoded classical signals with random numbers and combining the security analysis and key extraction method. Besides the point-to-point communication, EAQC can also be extended to build an entanglement-assisted metropolitan quantum communication network with the help of quantum routers and data centers. Furthermore, by connecting the metropolitan networks, a wide-area entanglement-assisted quantum communication network in a mesh topology can be established^45–48^.

In summary, we realize the deterministic EAQC based on the CV EPR entangled state over 20 km fiber channel by using the improved classical signals in the encoding process and reducing the excess noise in the fiber channel. With the help of the CV entanglement and FDM, 10 encoded weak classical signals, which can not be retrieved by coherent state, are extracted simultaneously. We show that the channel capacity of the deterministic EAQC at different transmission distances are enhanced significantly compared to that of the coherent state. Our results represent a milestone for realizing the deterministic EAQC with CV system in fiber channels and lay the foundation to the construction of entanglement-assisted quantum communication networks in the future.

## Materials and methods

### Details of the experiment

In the experiment, we use a narrow linewidth fiber laser (E15, NKT Photonics) at 1550 nm as the laser source. The laser beam is divided into three parts, which are used for the generation of the second harmonic beam, the seed beam of the OPA cavity and the coherent beam. The coherent beam is coupled into the fiber and separated by a fiber 99:1 beamsplitter, where 1% of the coherent beam is used as the ancilla beam (about 1 uW) to realize the encoding and 99% of the coherent beam is used as the local beam transmitted to Bob.

The second harmonic generation (SHG) cavity, which is designed as the semi-monolithic structure, consists of a 10 mm PPKTP crystal and a concave mirror. The convex face of the PPKTP crystal has the curvature radius of 12 mm and is coated with a high reflectivity for both 1550 nm and 775 nm. The plane face of the crystal is anti-reflectively coated for both 1550 nm and 775 nm to reduce the intra-cavity loss. The concave mirror (curvature radius equals to 25 mm) has the coating parameters of *T* = 12.5% at 1550 nm and *R* < 0.01% at 775 nm and serves as the input and output coupler simultaneously. The total conversion efficiency of the SHG cavity from 1550 nm to 775 nm is around 80% when the injected power of the fundamental beam increases to 1 W.

The structure of the OPA cavity is the same with the SHG cavity except for the coating parameters. The convex face of the OPA crystal is coated as *T* = 20% at 775 nm and high reflectivity at 1550 nm, which serves as the input coupler of the OPA cavity. The output coupler has the coating parameters of *T* = 12.5% at 1550 nm and high reflectivity at 775 nm. The optical length of the OPA cavity is 82 mm, resulting in the finesse of 50 at 1550 nm and 31 at 775 nm.

The InGaAs photodiode with a bandwidth of 35 MHz customized from Laser Components in Germany is used in our experiment. It is AR-coated with 20°s-polarized at 1550 nm. The reflected light can be reflected back to the photodiode by a concave mirror to increase the collection efficiency. Thus, more than 99% quantum efficiency of the photodiode at 1550 nm is promised. The ratio between the variance of the output of the detector measured at the high local oscillator power and zero local oscillator (electronic noise) is named shot noise clearance, which represents the efficiency of the homodyne detector. In our experiment, a 2 mW local beam is applied to the homodyne detector, resulting in the shot noise clearance of 20 dB, which corresponds to the loss of 1%. Therefore, the detection efficiency of 98% is obtained in our experiment, including the quantum efficiency of the photodiode (99%) and the efficiency of the homodyne detector (99%).

### The SNR in the EAQC

As shown in Fig. 1, the CV EPR entangled state with modes ${\hat{b}}_{1}$ and ${\hat{b}}_{2}$ is obtained by coupling two amplitude squeezed states ${\hat{a}}_{1}$ and ${\hat{a}}_{2}$ on a 50:50 beamsplitter, which are written as

4 $$\begin{array}{rcl}{\hat{a}}_{1} & = & \frac{1}{2}\left({e}^{-r}\,{\hat{x}}^{0}+i{e}^{r}\,{\hat{p}}^{0}\right)\\ {\hat{a}}_{2} & = & \frac{1}{2}\left({e}^{-r}\,{\hat{x}}^{0}+i{e}^{r}\,{\hat{p}}^{0}\right)\end{array}$$

where $\hat{x}=({\hat{a}}^{\dagger }+\hat{a})$ and $\hat{p}=i({\hat{a}}^{\dagger }-\hat{a})$ are the amplitude and phase quadratures of an optical mode $\hat{a}$ respectively, *r* (>0) is the squeezing parameter, and the superscript 0 represents the vacuum state whose variances are $V({\hat{x}}^{0})=V({\hat{p}}^{0})=1$ (corresponds to the SNL). The output beams of the beamplitter are given by

5 $$\begin{array}{rcl}{\hat{b}}_{1} & = & \frac{1}{\sqrt{2}}({\hat{a}}_{1}+i{\hat{a}}_{2})\\ {\hat{b}}_{2} & = & \frac{1}{\sqrt{2}}({\hat{a}}_{1}-i{\hat{a}}_{2})\end{array}$$

where *i* represents the relative phase difference of *π*/2 between modes ${\hat{a}}_{1}$ and ${\hat{a}}_{2}$. The quantum correlations of the CV EPR state have the forms of $V({\hat{x}}_{{b}_{1}}+{\hat{x}}_{{b}_{2}})=2{e}^{-2r}$ and $V({\hat{p}}_{{b}_{1}}-{\hat{p}}_{{b}_{2}})=2{e}^{-2r}$, which means that the amplitude (phase) quadratures of the EPR state are anti-correlated (correlated). We have $V({\hat{x}}_{{b}_{1}}+{\hat{x}}_{{b}_{2}})\to 0$ and $V({\hat{p}}_{{b}_{1}}-{\hat{p}}_{{b}_{2}})\to 0$ in the case of infinite squeezing (*r* → *∞*).

In the encoding process, Alice modulates the classical signals ${\hat{x}}_{s}$ and ${\hat{p}}_{s}$ on the amplitude and phase quadratures of the optical mode ${\hat{b}}_{1}^{E}$, we have ${\hat{x}}_{{b}_{1}^{E}}={\hat{x}}_{{b}_{1}}+{\hat{x}}_{s}$ and ${\hat{p}}_{{b}_{1}^{E}}={\hat{p}}_{{b}_{1}}+{\hat{p}}_{s}$ respectively. In the decoding process, Bob couples modes ${\hat{b}}_{1}^{E}$ and ${\hat{b}}_{2}$ on a 50:50 beamsplitter with the relative phase difference of 0, the output modes are given by

6 $$\begin{array}{rcl}{\hat{d}}_{1} & = & \frac{1}{\sqrt{2}}({\hat{b}}_{1}^{E}+{\hat{b}}_{2})\\ {\hat{d}}_{2} & = & \frac{1}{\sqrt{2}}({\hat{b}}_{1}^{E}-{\hat{b}}_{2})\end{array}$$

After the decoding process, Bob measures the variances of ${\hat{x}}_{{d}_{1}}$ and ${\hat{p}}_{{d}_{2}}$ by two homodyne detectors simultaneously. The amplitude quadrature of mode ${\hat{d}}_{1}$ and phase quadrature of mode ${\hat{d}}_{2}$ are given by

7 $$\begin{array}{rcl}{\hat{x}}_{{d}_{1}} & = & \frac{1}{\sqrt{2}}({\hat{x}}_{{b}_{1}}+{\hat{x}}_{{b}_{2}}+{\hat{x}}_{s})={\hat{x}}_{{a}_{1}}+\frac{1}{\sqrt{2}}{\hat{x}}_{s}\\ {\hat{p}}_{{d}_{2}} & = & \frac{1}{\sqrt{2}}({\hat{p}}_{{b}_{1}}-{\hat{p}}_{{b}_{2}}+{\hat{p}}_{s})={\hat{x}}_{{a}_{2}}+\frac{1}{\sqrt{2}}{\hat{p}}_{s}\end{array}$$

It is obvious that the encoded classical signals can be decoded simultaneously by measuring the amplitude quadrature of mode ${\hat{d}}_{1}$ and phase quadrature of mode ${\hat{d}}_{2}$ simultaneously.

According to Eqs. (4-7), the variances of the output modes are given by

8 $$\begin{array}{rcl}V({\hat{x}}_{{d}_{1}}) & = & \frac{1}{2}[V({\hat{x}}_{{b}_{1}}+{\hat{x}}_{{b}_{2}})+V({\hat{x}}_{s})]=V({\hat{x}}_{{a}_{1}})+\frac{1}{2}V({\hat{x}}_{s})\\ V({\hat{p}}_{{d}_{2}}) & = & \frac{1}{2}[V({\hat{p}}_{{b}_{1}}-{\hat{p}}_{{b}_{2}})+V({\hat{p}}_{s})]=V({\hat{x}}_{{a}_{2}})+\frac{1}{2}V({\hat{p}}_{s})\end{array}$$

In this case, the variances of decoded weak classical signals on amplitude and phase quadratures are $\frac{1}{2}V({\hat{x}}_{s})$ and $\frac{1}{2}V({\hat{p}}_{s})$, respectively. The corresponding noise of decoded amplitude and phase quadratures are $V({\hat{x}}_{{a}_{1}})$ and $V({\hat{x}}_{{a}_{2}})$, respectively. Thus, the SNRs obtained from Bob’s station are given by^39^

9 $$\begin{array}{rcl}SN{R}_{x} & = & \frac{\frac{1}{2}V({\hat{x}}_{s})}{V({\hat{x}}_{{a}_{1}})}=\frac{\frac{1}{2}V({\hat{x}}_{s})}{{e}^{-2r}}\\ SN{R}_{p} & = & \frac{\frac{1}{2}V({\hat{p}}_{s})}{V({\hat{x}}_{{a}_{2}})}=\frac{\frac{1}{2}V({\hat{p}}_{s})}{{e}^{-2r}}\end{array}$$

Considering the transmission loss and excess noise in the fiber channel, the output modes after decoding are given by

10 $$\begin{array}{rcl}{\hat{d}}_{1}^{{\prime} } & = & \frac{1}{\sqrt{2}}({\hat{b}}_{1}^{{E}^{{\prime} }}+{\hat{b}}_{2}^{{\prime} })\\ {\hat{d}}_{2}^{{\prime} } & = & \frac{1}{\sqrt{2}}({\hat{b}}_{1}^{{E}^{{\prime} }}-{\hat{b}}_{2}^{{\prime} })\end{array}$$

and the corresponding variances of output modes ${\hat{d}}_{1}^{{\prime} }$ and ${\hat{d}}_{2}^{{\prime} }$ are expressed as

11 $$\begin{array}{rcl}V({\hat{x}}_{{d}_{1}^{{\prime} }}) & = & \frac{1}{2}\eta V({\hat{x}}_{s})+\eta V({\hat{x}}_{{a}_{1}})+\\ & & \frac{1}{2}(1-\eta )[V({\hat{x}}_{{\nu }_{1}})+V({\hat{x}}_{{\nu }_{2}})+{\delta }_{1}+{\delta }_{2}]\\ V({\hat{p}}_{{d}_{2}^{{\prime} }}) & = & \frac{1}{2}\eta V({\hat{p}}_{s})+\eta V({\hat{x}}_{{a}_{2}})+\\ & & \frac{1}{2}(1-\eta )[V({\hat{p}}_{{\nu }_{1}})+V({\hat{p}}_{{\nu }_{2}})+{\delta }_{1}+{\delta }_{2}]\end{array}$$

where $V({\hat{x}}_{{\nu }_{1}})=V({\hat{x}}_{{\nu }_{2}})=V({\hat{p}}_{{\nu }_{1}})=V({\hat{p}}_{{\nu }_{2}})=1$ are the variances of the vacuum states. In Eq. (11), the variances of decoded signals are $\frac{1}{2}\eta V({\hat{x}}_{s})$ and $\frac{1}{2}\eta V({\hat{p}}_{s})$, the corresponding variances of noise background are $V{({\hat{x}}_{{d}_{1}^{{\prime} }})}_{N}=\eta V({\hat{x}}_{{a}_{1}})+\frac{1}{2}(1-\eta )[V({\hat{x}}_{{\nu }_{1}})+V({\hat{x}}_{{\nu }_{2}})+{\delta }_{1}+{\delta }_{2}]$ and $V{({\hat{p}}_{{d}_{2}^{{\prime} }})}_{N}=\eta V({\hat{x}}_{{a}_{2}})+\frac{1}{2}(1-\eta )[V({\hat{p}}_{{\nu }_{1}})+V({\hat{p}}_{{\nu }_{2}})+{\delta }_{1}+{\delta }_{2}]$ respectively. Thus, the SNRs at Bob’s station are given by

12 $$\begin{array}{rcl}SN{R}_{x}^{{\prime} } & = & \frac{\frac{1}{2}\eta V({\hat{x}}_{s})}{V{({\hat{x}}_{{d}_{1}^{{\prime} }})}_{N}}\\ SN{R}_{p}^{{\prime} } & = & \frac{\frac{1}{2}\eta V({\hat{p}}_{s})}{V{({\hat{p}}_{{d}_{2}^{{\prime} }})}_{N}}\end{array}$$

which are the results presented in Eq. (3).

## Supplementary information

Supplementary Information

## Data Availability

All data needed to evaluate the conclusions in the paper are present in the paper and/or the Supplementary Information.
