# Supplementary material for: Deterministic entanglement-assisted quantum communication over 20 km fiber channel
Source: Light Sci Appl. 2026 Jan 23;15:83. doi: 10.1038/s41377-025-02173-6 (PMC12830655; doi:10.1038/s41377-025-02173-6)
Supplement: Supplementary file 1 — Supplementary Information [file 41377_2025_2173_MOESM1_ESM.pdf]

Supplementary Information for
Deterministic entanglement-assisted quantum communication over 20-km fiber channel

Siyu Ren[1,†], Yanru Yan[1,†], Yalin Li[1], Chao Li[1], Dongmei Han[1], Xuezhi Zhu[1], Meihong Wang[1,2], and Xiaolong Su[1,2,*]

[1] *State Key Laboratory of Quantum Optics Technologies and Devices, Institute of Opto-Electronics, Shanxi University, Taiyuan, 030006, China*

[2] *Collaborative Innovation Center of Extreme Optics, Shanxi University, Taiyuan, 030006, China*

[*] *Correspondence: Xiaolong Su (suxl@sxu.edu.cn)*

[†] *These authors contributed equally: Siyu Ren, Yanru Yan*

S1. Note S1: Details of locking systems

In our experiment, both the cavity length of optical parametric amplifiers (OPA) and several relative phase difference need to be controlled, which involves nine locking systems, as shown in Fig. S1. Locking systems 1 and 2 are used to control the length of two OPA cavities respectively, where the Pound-Drever-Hall (PDH) locking technique is applied. A 55 MHz modulation signal is added on the optical path of OPA's seed beam by an electronic-optical modulator. The error signal is extracted by mixing the output of the photo-detector and the reference signal at 55 MHz, and is fed back to a piezo mounted on the concave mirror of the OPA cavity after passing through a proportional integral derivative (PID) module and a high voltage amplifier.

Locking systems 3 and 4 are used to lock the relative phase difference between the seed beam and the pump beam to OPA cavity. Locking system 6 is used to lock the phase difference between one of the entangled beams and the ancilla beam (encoding process). These three locking systems are based on the FPGA modules (Redpitaya-STEMlab125-14) to realize the phase locking of 0 or π. The modulation signal around 30 kHz generated by the FPGA module is added

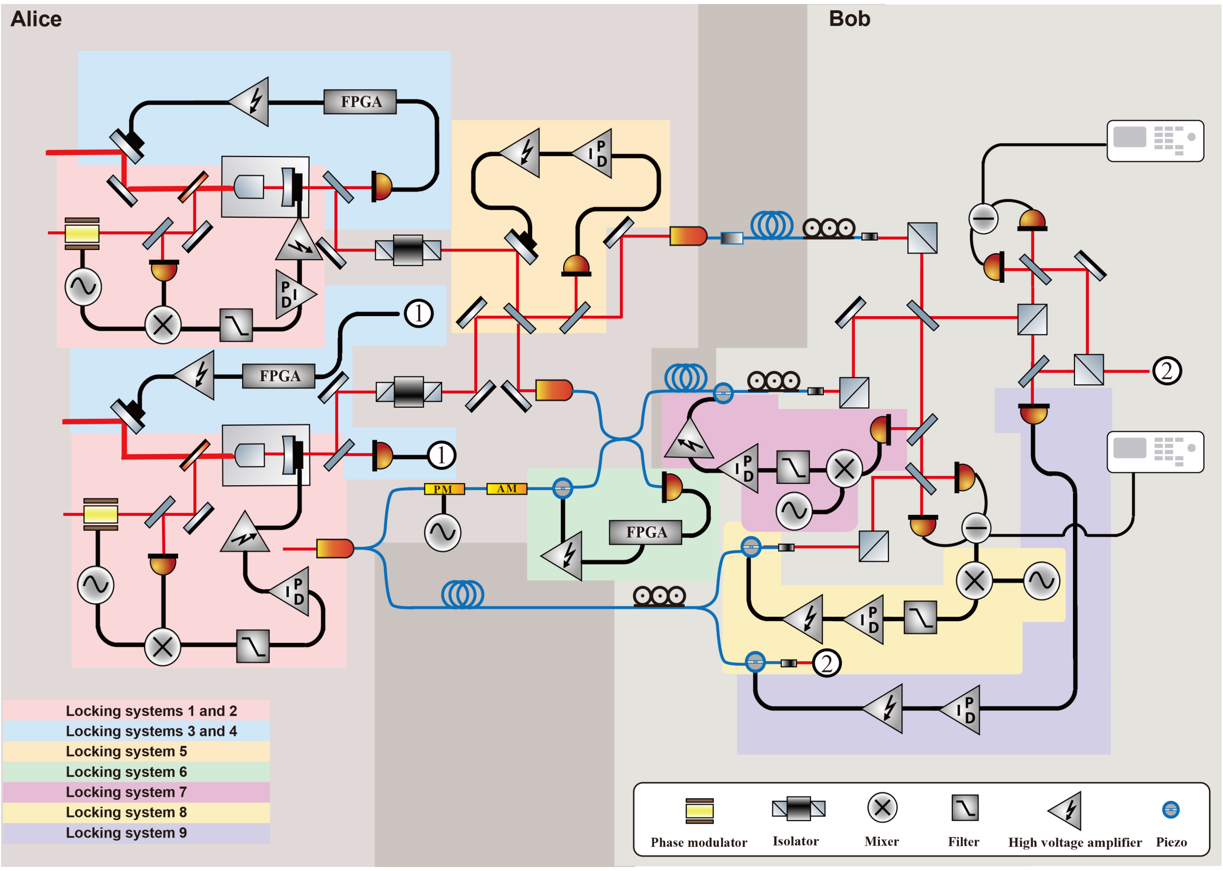

FIG. S1. **Schematic of locking systems in the experiment.**

to the piezo in the optical path. The error signal is obtained by mixing and filtering of the detected interference signal in the FPGA module and is fed back to the piezo in the optical path and/or fiber stretcher after passing through a high voltage amplifier. Locking systems 5 and 9 are used to lock the relative phase differences of $\pi/2$. In this case, only a PID module and a high voltage amplifier are applied to control the relative phase differences since the interference signal itself can be regarded as the error signal.

In our experiment, the most challenging part is the phase locking of optical beams transmitted after the fiber channels (Locking systems 7 and 8). Due to the random phase drift in independent fibers, the relative phase changes over time. Here, a 6 MHz modulation signal is added on the encoded beam by a fiber electronic-optical modulator. The error signal is extracted by mixing the output of the photo-detector (locking system 7) or homodyne detector (locking system 8) with the reference signal at 6 MHz, and is fed back to a fiber stretcher (PZT1-LSM-PMF1-FA-10B) via a PID module and a high voltage amplifier. By optimizing the PID parameters, the phase fluctuation is reduced to 2.45° at 20.121 km.

S2. Note S2: The phase fluctuation in the experiment

A. Note S2.1: The effect of phase fluctuation on measurement results

Since the EPR beams and the local beam transmitted in independent fibers, the phase fluctuation occurs in the phase locking between modes $\hat{b}'_1$ and $\hat{b}'_2$, as well as the phase locking between the local beam and $\hat{d}'_1$ ($\hat{d}'_2$). Taking the effect of phase fluctuation on the noise background into account, the output beams after decoding are expressed as

$$\begin{aligned}\hat{d}''_1 &= \frac{1}{\sqrt{2}}(\hat{b}'_1 + \hat{b}'_2 e^{i(\theta+\Delta\theta_1)}) \\ \hat{d}''_2 &= \frac{1}{\sqrt{2}}(\hat{b}'_1 - \hat{b}'_2 e^{i(\theta+\Delta\theta_1)})\end{aligned} \tag{S1}$$

where $\theta = 0$ represents the relative phase between modes $\hat{b}'_1$ and $\hat{b}'_2$, $\Delta\theta_1$ represents the corresponding phase fluctuation.

In the analysis for effect of phase fluctuation on homodyne detection, we take the measurement of amplitude quadrature for mode $\hat{v}$ as an example. In this case, mode $\hat{v}$ is coupled with the local beam $\hat{L}$ on a 50:50 beam splitter and the relative phase difference is locked to 0. The output modes $\hat{m}$ and $\hat{n}$ of the 50:50 beam splitter are expressed by

$$\begin{aligned}\hat{m} &= \frac{1}{\sqrt{2}}(\hat{v} + \hat{L}e^{i(0+\Delta\theta)}) \\ \hat{n} &= \frac{1}{\sqrt{2}}(\hat{v} - \hat{L}e^{i(0+\Delta\theta)})\end{aligned} \tag{S2}$$

The photocurrents of two photodiodes are proportional to photon numbers of modes $\hat{m}$ and $\hat{n}$,

$$\begin{aligned}i_m &= \hat{m}^\dagger\hat{m} = \frac{1}{2}(\hat{v}^\dagger\hat{v} + \hat{v}^\dagger\hat{L}e^{i\Delta\theta} + \hat{L}^\dagger\hat{v}e^{-i\Delta\theta} + \hat{L}^\dagger\hat{L}) \\ i_n &= \hat{n}^\dagger\hat{n} = \frac{1}{2}(\hat{v}^\dagger\hat{v} - \hat{v}^\dagger\hat{L}e^{i\Delta\theta} - \hat{L}^\dagger\hat{v}e^{-i\Delta\theta} + \hat{L}^\dagger\hat{L})\end{aligned} \tag{S3}$$

Thus, the output photocurrent of the homodyne detector is given by

$$i = i_m - i_n = \hat{v}^\dagger\hat{L}e^{i\Delta\theta} + \hat{L}^\dagger\hat{v}e^{-i\Delta\theta} \tag{S4}$$

By rewriting the modes $\hat{v}$ and $\hat{L}$ in terms of linearized operators $\hat{v} = u + \delta\hat{v}$, $\hat{L} = l + \delta\hat{l}$, Eq. (S4) is expressed as

$$i = u^* l e^{i\Delta\theta} + l^* u e^{-i\Delta\theta} + u^*\delta\hat{L}e^{i\Delta\theta} + u\delta\hat{L}e^{-i\Delta\theta} + l\delta\hat{v}^\dagger e^{i\Delta\theta} + l^*\delta\hat{v}e^{-i\Delta\theta} \tag{S5}$$

The first and second terms in Eq. (S5) represent the mean value of the photocurrent. In the homodyne detection, when the power of local beam is much higher than that of signal beam ($l \gg u$), the third and fourth terms in Eq. (S5) are ignored. Thus, the output photocurrent of the homodyne detector related to the fluctuation of mode $\hat{v}$ is expressed by

$$\begin{aligned}\delta_i &= l\delta\hat{v}^\dagger e^{i\Delta\theta} + l^*\delta\hat{v}e^{-i\Delta\theta} \\ &= l(\delta\hat{v}^\dagger e^{i\Delta\theta} + \delta\hat{v}e^{-i\Delta\theta})\end{aligned} \tag{S6}$$

where

$$\begin{aligned}\delta\hat{v}^{\dagger}e^{i\Delta\theta}+\delta\hat{v}e^{-i\Delta\theta} &= \delta\hat{v}^{\dagger}(\cos(\Delta\theta)+i\sin(\Delta\theta))+\delta\hat{v}(\cos(\Delta\theta)-i\sin(\Delta\theta)) \\ &= \cos(\Delta\theta)(\delta\hat{v}+\delta\hat{v}^{\dagger})+\sin(\Delta\theta)(i\delta\hat{v}^{\dagger}-i\delta\hat{v}) \\ &= \cos(\Delta\theta)(\delta\hat{x}_v)+\sin(\Delta\theta)(\delta\hat{p}_v) \end{aligned} \tag{S7}$$

In this case, the variance of the amplitude quadrature for $\hat{v}$ considering the phase fluctuation $\Delta\theta$ is expressed by

$$V(\hat{x}_v)' = V(\hat{x}_v)\cos^2(\Delta\theta)+V(\hat{p}_v)\sin^2(\Delta\theta) \tag{S8}$$

The influence of phase fluctuation on the phase quadrature measurement in the homodyne detection can be obtained in the same way, which is given by

$$V(\hat{p}_v)' = V(\hat{p}_v)\cos^2(\Delta\theta)+V(\hat{x}_v)\sin^2(\Delta\theta) \tag{S9}$$

Therefore, the obtained variances for the noise background of output modes $\hat{d}_1''$ and $\hat{d}_2''$ in the homodyne detection considering the phase fluctuation are given by

$$\begin{aligned} V(\hat{x}_{d_1''})'_N &= V(\hat{x}_{d_1''})_N\cos^2\Delta\theta_2+V(\hat{p}_{d_1''})_N\sin^2\Delta\theta_2 \\ V(\hat{p}_{d_2''})'_N &= V(\hat{p}_{d_2''})_N\cos^2\Delta\theta_3+V(\hat{x}_{d_2''})_N\sin^2\Delta\theta_3 \end{aligned} \tag{S10}$$

where $V(\hat{x}_{d_1''})_N[V(\hat{p}_{d_1''})_N]$, $V(\hat{x}_{d_2''})_N[V(\hat{p}_{d_2''})_N]$ are the variances for the noise background of $\hat{d}_1''$ and $\hat{d}_2''$, respectively. $\Delta\theta_2$ ($\Delta\theta_3$) represents the phase fluctuation in the measurement of amplitude (phase) quadrature. Since the improved classical signals are applied in the encoding process, the SNRs that considering the phase fluctuation are expressed by

$$\begin{aligned} SNR_x'' &= \frac{1}{V(\hat{x}_{d_1''})'_N} \\ SNR_p'' &= \frac{1}{V(\hat{p}_{d_2''})'_N} \end{aligned} \tag{S11}$$

B. Note S2.2: Measured phase fluctuation in the experiment

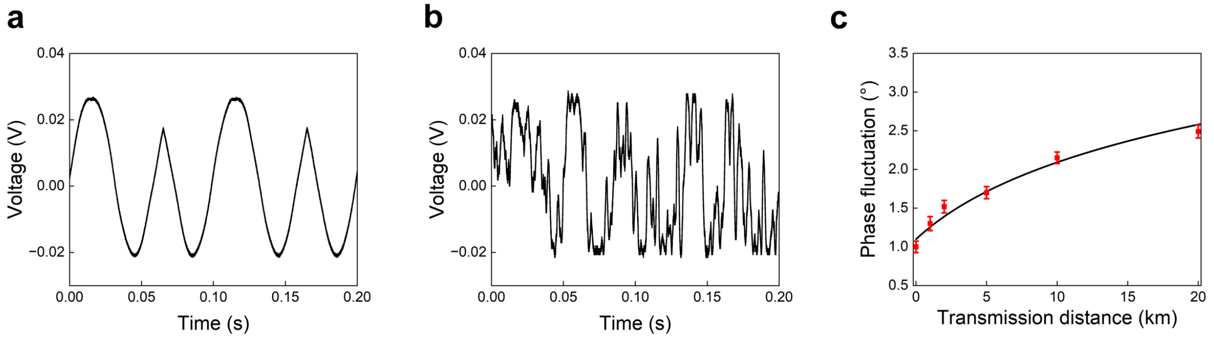

FIG. S2. **Phase fluctuation in the experiment.** **a** and **b** Interference patterns between two coherent beams transmitted in independent fibers channels of 0.002 km and 5 km respectively when the relative phase is scanned. **c** The dependence of phase fluctuation on transmission distance.

To present the influence of phase fluctuation, the interference patterns between two coherent beams transmitted in independent fibers at different transmission distances are shown in Fig. S2a and Fig. S2b. When the transmission distance is limited to 0.002 km, a normal interference pattern is obtained. When we extend the transmission distance to 5 km, the messy interference pattern is observed, which brings the challenge to the phase locking system. This problem is solved by optimizing the locking systems for optical beams transmitted after the fiber channels, as presented in Section S1.

The measured phase fluctuation of phase locking system at different transmission distances is shown in Fig. S2c. Based on the measurement results (red data), a fitted curve of phase fluctuation at different transmission distances that suitable for the current experimental system is obtained. The corresponding fitting function is given by

$$\Delta\theta = \ln(3 + 0.51 * L) \tag{S12}$$

where $\Delta\theta$ is the phase fluctuation and L is the fiber length. Since $\Delta\theta_1 \approx \Delta\theta_2 \approx \Delta\theta_3 = \Delta\theta$ in our experiment, by substituting Eq. (S12) into Eqs. (S10), the dependence of channel capacity on transmission distance is obtained according to Eqs. (S11) and (1), as shown in Fig. 6 in the Main text.

S3. Note S3: Supplemental measurement results

We measure the dependence of signal-to-noise ratio on transmission distance when the fixed classical signals are encoded at Alice's station, as shown in Fig. S3. It is obvious that the decoded classical signals decrease with the increased transmission distance. When the transmission distance reaches 10 km, the decoded classical signals are completely submerged in the noise background and cannot be retrieved, even though the noise background at Bob's station remains lower than the shot noise limit. Therefore, using fixed-size classical signals reduces the communication distance.

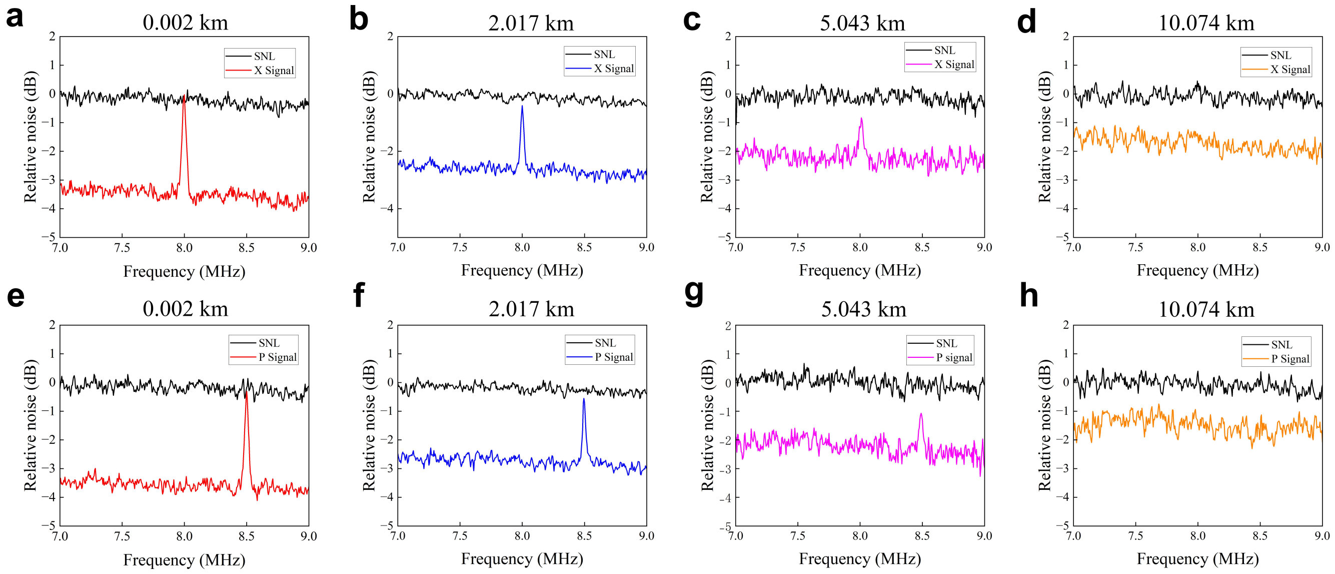

FIG. S3. **Measurement results of the deterministic entanglement-assisted quantum communication in the fiber channel when the fixed classical signals are encoded at Alice's station.**

We also compare the channel capacities obtained using improved classical signals (Scheme 1) and fixed classical signals (Scheme 2), as presented in TABLE S1. Since the encoded signals attenuate with the increased loss, the channel capacity of Scheme 2 significantly decreases compared to Scheme 1 with longer transmission distances. The channel capacity can not be calculated when the transmission distance reaches 10 km since the encoded signals are completely submerged in the noise background. In this case, the advantage of the entangled state disappears. Thus, the improved classical signals chosen in our experiment are beneficial to enhance the channel capacity and extend the transmission distance.

TABLE S1. **The channel capacities of deterministic entanglement-assisted quantum communication in the fiber channel with improved classical signals (Scheme1) and fixed classical signals (Scheme 2).**

Channel capacity (bits)	Transmission distance (km)				
	0.002	2.017	5.043	10.074	20.121
scheme 1	1.73	1.59	1.49	1.32	1.16
scheme 2	1.72	1.46	1.24	-	-

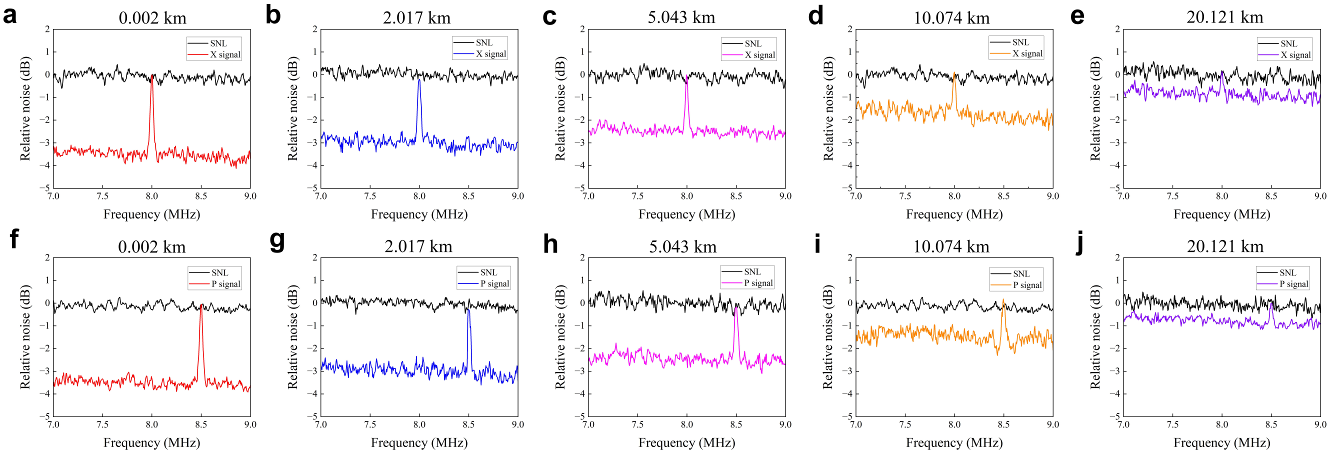

FIG. S4. **Measurement results of the deterministic entanglement-assisted quantum communication with improved classical signals in the fiber channel.**

S4. Note S4: The dependence of channel capacity on average photon numbers

The average photon number of the optical beam carrying the classical signal is given by [1]

$$n = \frac{1}{4}[V_x + V_p + V(x_s) + V(p_s)] - \frac{1}{2} \tag{S13}$$

where $V(x_s)$ and $V(p_s)$ are the variances of the classical signals, V_x and V_p are the variances of amplitude and phase quadratures of the quantum state. In order to embody the advantage of quantum dense coding, the improved weak classical signals $V(x_s) = V(p_s) = 2/\eta$ are applied in the encoding process in our experiment, which can be regarded as normalizing the encoded classical signals to the sender (Alice). In this case, we have $V(x_s) = V(p_s) = 2$ at Bob's station at different transmission distances. The variance $V_x(V_p)$ equals to 5.28 (4.92), 4.21 (4.41), 3.60 (3.72), 3.42 (3.24), and 2.79 (2.82) at the transmission distances of 0.002, 2.017, 5.043, 10.074, and 20.121 km according to the measurement results in Figs. 4a and 4b in the main text. Therefore, according to Eq. (S13), we have the average photon number n_{qd} equals to 3.05, 2.65, 2.33, 2.16, and 1.90, as shown in TABLE S2. By applying frequency division multiplexing (FDM), the average photon number (n_{qd-FDM}) is further increased to 7.05, 6.65, 6.33, 6.16, and 5.90 at five transmission distances. The channel capacity (C_{qd-FDM}) of dense coding with the entangled state is also increased at each distance, as shown in TABLE S2.

TABLE S2. **Average photon numbers and channel capacities at different transmission distances with the coherent state and the entangled state, respectively.**

Distance (km)	n_{coh}	n_{qd}	C_{coh}	C_{qd}	$n_{coh-FDM}$	n_{qd-FDM}	$C_{coh-FDM}$	C_{qd-FDM}
0.002	1	3.05	1	1.73	5	7.05	1	3.66
2.017	1	2.65	1	1.59	5	6.65	1	3.44
5.043	1	2.33	1	1.49	5	6.33	1	3.35
10.074	1	2.16	1	1.32	5	6.16	1	3.05
20.121	1	1.9	1	1.16	5	5.90	1	2.83

Please note that for dense coding with the coherent state, we have $V_x = V_p = 1$. Thus, the average photon number n_{coh} is always equal to 1 at different transmission distances. If the entangled state is replaced by the coherent state, no signal can be retrieved since the improved weak classical signals are encoded in the experiment, which results in the channel capacity of using the coherent state (C_{coh}) always equal to 1 at different transmission distances. When FDM is applied in the experiment, the average photon number with coherent state $(n_{coh-FDM})$ is increased. However, the channel capacity of dense coding with coherent state $(C_{coh-FDM})$ is still equal to 1 since no signals can be retrieved with the coherent state when the improved weak signals are encoded at Alice's station.

In the case of comparing the channel capacity of different schemes with the same average photon number, the classical signal $V(x_s) = V(p_s) = V_s$ is regarded as a variable parameter which can be obtained according to Eq. (S13). Thus, the SNRs are obtained by

$$SNR_x = SNR_p = \frac{S}{N} = \frac{\frac{1}{2}V_s}{V_{sq}} = \frac{\frac{1}{4}(4n + 2 - V_x - V_p)}{V_{sq}} \tag{S14}$$

where $\frac{1}{2}$ represents that the classical signal V_s is divided into two parts by the 50:50 beamsplitter in the decoding process and V_{sq} is the variance of the squeezed state after decoding at Bob's station (for dense coding with the coherent state, we have $V_{sq} = 1$). Thus, the dependence of channel capacity on average photon number is given by

$$C = \frac{1}{2}\log_2(1 + SNR_x) + \frac{1}{2}\log_2(1 + SNR_p) = \log_2[1 + \frac{\frac{1}{4}(4n + 2 - V_x - V_p)}{V_{sq}}] \tag{S15}$$

According to the measurement results in Figs. 4c, 4d, 5b, and 5c in the main text, we have V_{sq} equals to around -3.65, -3.05, -2.60, -1.77, and -0.94 dB at the transmission distance (L) of 0.002, 2.017, 5.043, 10.074, and 20.121 km respectively, which corresponds to the variances V_{sq} of 0.43, 0.50, 0.55, 0.67, and 0.81 at the five transmission distances. According to the measurement results in Figs. 4a and 4b in the main text, we have $V_x(V_p)$ equals to 5.28 (4.92), 4.21 (4.41), 3.60 (3.72), 3.42 (3.24), and 2.79 (2.82) at the five transmission distances. By substituting the variances of V_x, V_p, and V_{sq} at each transmission distance to Eq. (S15) respectively, we obtain the dependence of channel capacities on average photon number at different transmission distances, as shown by the gray solid, black solid, cyan solid, pink solid, and blue solid curves in Fig. 6b in the main text.

---

[1] Braunstein, S. L. & Kimble, H. J. Dense coding for continuous variables. *Physical Review A* **61**, 042302 (2000).
